# Exploring the benefits of traditional Chinese exercises (Tai Chi and Qigong) on the anxiety and depression of older adults: A systematic review and meta-analysis

**DOI:** 10.1097/MD.0000000000041908

**Published:** 2025-03-21

**Authors:** Yangjian Dong, Dan Pang, Jie Xiang, Guodong Chao, Xiaoqin Kuang

**Affiliations:** a College of Physical Education, China Three Gorges University, Yichang, China; b College of Physical Education, Hubei Preschool Teachers College, Wuhan, China; c College of Physical Education and Health, Guilin University, Guilin, China.

**Keywords:** anxiety, depression, meta-analysis, traditional Chinese exercises

## Abstract

**Background:**

Research shows that traditional Chinese exercises (TCEs) significantly improve anxiety and depression in older adults. However, studies on the effects of different exercise durations, frequencies, and intensities in this population are limited. This systematic review and meta-analysis evaluate the impact of TCEs on anxiety and depression, and explores the optimal exercise parameters, aiming to provide evidence for nonpharmacological treatment options in clinical practice.

**Methods:**

As of August 2023, we conducted a literature search through 3 English electronic databases to identify relevant studies. We included studies that met our criteria. During the literature inclusion process, we used Review Manager 5.4 to create flow diagrams, assess the risk of bias, and perform statistical analyses.

**Results:**

A total of 31 eligible studies involving 2501 participants were included. Compared with the control group, TCEs showed significant improvements in anxiety (standardized mean differences [SMD] = −0.93, 95% confidence interval [CI]: −1.78 to −0.08, *P* = .03, *I*^2^ = 96%) and depression (SMD = −1.14, 95% CI: −1.82 to −0.47, *P* = .03, *I*^2^ = 96%). Subgroup analyses indicated that an intervention duration of 12 to 16 weeks yielded the largest effect size for anxiety (SMD = −1.36, 95% CI: −2.36 to −0.36, *P* = .008), while the 24-week group showed the largest effect size for depression (SMD = −0.87, 95% CI: −1.43 to −0.30, *P* = .002). For intervention frequency, a regimen of 3 to 4 times per week produced the largest effect size for anxiety (SMD = −2.34, 95% CI: −4.69 to 0.02, *P* = .05), whereas a frequency of 5 to 7 times per week demonstrated the largest effect size for depression (SMD = −1.00, 95% CI: −1.83 to −0.17, *P* = .02). Regarding single-session exercise duration, a group exercising for 40 to 60 minutes showed the largest effect sizes for anxiety (SMD = −1.38, 95% CI: −2.40 to −0.37, *P* = .007) and depression (SMD = −0.75, 95% CI: −1.07 to −0.42, *P* < .00001).

**Conclusion:**

The results indicate that TCEs significantly alleviate anxiety and depression in older adults, with intervention frequency, intensity, and duration potentially influencing the outcomes. However, heterogeneity across studies was observed, primarily due to differences in intervention types and control group designs. These findings offer valuable guidance for future research directions.

## 1. Introduction

The World Health Organization reports that mental health conditions significantly affect the incidence and outcomes of other health issues.^[1–3]^ According to the 2019 Global Burden of Disease Report, mental disorders are the second leading cause of disease burden worldwide.^[4,5]^ Depression ranks 2nd and anxiety 8th among the top 25 causes of reduced healthy life expectancy.^[6]^ Depression represents a significant global health issue, affecting disability-adjusted life years; 2nd only to heart disease.^[7,8]^ While anxiety disorders can affect all age groups, they are particularly severe in older adults, where they have become widespread. Factors such as lack of support from family and friends, along with adverse social and environmental conditions, can worsen stress and anxiety in this population. These conditions place a significant burden on public health systems, making it essential to reduce their prevalence to address public health challenges.^[9,10]^

The primary treatments for anxiety and depression are pharmacological interventions and cognitive therapy. However, pharmacological treatments can lead to dependency and adverse effects, increasing long-term economic costs.^[11]^ Cognitive therapy, on the other hand, requires in-person sessions, which many patients avoid due to feelings of shame and the cost of treatment. Older adults, in particular, face additional challenges in accessing psychological therapy due to age-related and psychological factors. Given the limitations of these conventional treatments, physical exercise is gaining popularity as an alternative for managing anxiety and depression. Traditional Chinese exercises (TCEs), such as Tai Chi and Qigong (which includes Ba Duan Jin, Wu Qin Xi, Yi Jin Jing, and Liu Zi Jue), are low-intensity forms of physical activity that combine exercise, breathing techniques, and mindfulness. Compared with traditional medical interventions or pharmacological treatments, these exercises, with their low intensity and simple techniques, make it easier for older adults to adhere to the practice, offering unique benefits for enhancing physical and mental well-being.^[12,13]^ Studies have shown that TCEs have significant effects in relieving stress and depression, as well as improving sleep quality in old adults.^[14,15]^ In recent years, the number of randomized-controlled trials on the impact of Tai Chi and Qigong on the mental health of old adults has increased significantly.

However, there is currently a lack of systematic evaluation on the effects of TCEs at different times, frequencies, and intensities in improving anxiety and depression in old adults. Therefore, this study will follow the patient, intervention, comparison, outcome principle, integrate relevant literature, conduct a systematic review, and employ meta-analysis methods to comprehensively analyze all included data from randomized controlled trials to enhance the reliability of evidence. In addition, this study aimed to explore the effects of different exercise durations, frequencies, and intensities on anxiety and depression in old adults, thereby providing more precise strategies for clinical nonpharmacological treatment plans.

## 2. Methods

This study was conducted following the Preferred Reporting Items for Systematic Reviews and Meta-Analyses guidelines and the Cochrane Intervention Review Manual, while also being registered in the PROSPERO database with registration number CRD42023432254.

### 2.1. Search strategy

We searched the PubMed, Cochrane, and Web of Science databases, with English language restriction, from the inception of the databases until August 31, 2023. The search keywords were determined according to the patient, intervention, comparison, outcome principle, covering old adults, TCEs, traditional exercises, mind–body exercises, Qigong, Tai Chi, Ba Duan Jin, Wu Qin Xi, Yi Jin Jing, Liu Zi Jue, anxiety, and depression. In addition, manual searches were performed on the references of the included literature to supplement the retrieval of relevant literature. For a detailed search strategy, please refer to Supplementary File S1, http://links.lww.com/MD/O549.

### 2.2. Study selection

Two reviewers (YD and DP) read, systematically evaluated, and extracted information from the included literature. These 2 reviewers independently assessed the titles and abstracts of the literature and finally included the literature that met the selection criteria for full-text reading. In case of disagreement, they rechecked the original text or discussed it with a third reviewer (XK) and resolved any discrepancies after reaching a consensus.

The inclusion criteria are as follows: Only eligible randomized-controlled trials are included; participants are old adults (60 years or older); the intervention measures include Tai Chi, Qigong, Wu Qin Xi, Ba Duan Jin, or Yi Jinjing; the control group includes no exercise, daily activities, health education, medication, or low-intensity exercise; the literature should include outcome measures such as anxiety and depression.

The exclusion criteria are as follows: Exclusion of experiments with unscientific and rigorous research designs; exclusion of nonrandomized-controlled trials, case reports, and conference abstracts; exclusion of literature that mixes intervention measures with other activities; exclusion of literature with incomplete data.

### 2.3. Data extraction

Two reviewers (YD and DP) independently extracted data from the included articles. The extracted data included study characteristics (such as title, authors, publication year, use of randomization, allocation concealment, blinding method, and control group), participant characteristics (such as age and sample size), description of intervention measures (such as type of exercise, frequency, and duration), as well as intervention measures for the control group, and data and measurement tools related to anxiety and depression. We contacted the authors of the studies via email to obtain any missing information.

### 2.4. Assessment of risk bias

All the studies included in this research are randomized-controlled trials, and we utilized the Cochrane risk of bias assessment tool^[16]^ to assess the methodological quality of these trials. The assessment concentrated on various key aspects, including random sequence generation, allocation concealment, blinding of participants and personnel, blinding of outcome assessors, completeness of outcome data, selective reporting, and other biases. Two independent reviewers (JX and GC) conducted this evaluation, categorizing the quality domains as either high risk, low risk, or unclear risk. The criteria for evaluation were as follows: trials that lack clear descriptions of random sequence generation and allocation concealment were classified as high risk. Trials that did not implement blinding and outcome assessment due to the intervention nature were considered unclear risk; trials that did not report follow-up losses and data exclusions were deemed high risk, while those that omitted this information were regarded as unclear risk. Trials without selective reporting were labeled as low risk and trials without other potential sources of bias were also classified as low risk. Disagreements between 2 reviewers are resolved through discussion and, if necessary, consultation with a third independent reviewer (XK) to ensure a fair and consistent evaluation.

### 2.5. Data analysis

We utilized Review Manager 5.4 (a software developed by the Nordic Cochrane Centre for creating and maintaining Cochrane systematic reviews, and designed and updated by the International Cochrane Collaboration) to create literature flow diagrams, conduct Cochrane risk of bias assessments, and perform statistical analyses. Given that all variables in the studies were continuous and multiple measurement scales were used, we analyzed the results using standardized mean differences (SMD). The SMD is the difference between 2 means divided by the pooled standard deviation, which not only eliminates the impact of absolute values in individual studies but also removes the influence of measurement units on the outcomes. Therefore, this metric is particularly suitable for analyzing data with different units or large mean differences, along with 95% confidence intervals (CIs). A *P* value of <.05 indicated a significant effect, suggesting a notable difference between the experimental and control groups, while a *P* < .01 indicated a highly significant difference.

We assessed heterogeneity between studies using the *I*^2^ statistic. Heterogeneity refers to the variability or differences between studies included in the same meta-analysis. An *I*^2^ value of 0 indicates no heterogeneity, while *I*^2^ > 50% suggests significant heterogeneity. In the absence of heterogeneity, a fixed-effect model was used, while a random-effects model was applied when heterogeneity was present. Publication bias was evaluated using funnel plots. If studies were evenly distributed on both sides of the funnel plot, we concluded there was no publication bias; otherwise, publication bias was considered present. We also conducted sensitivity analyses by sequentially excluding each study to evaluate the robustness of the results. In addition, subgroup analyses based on intervention characteristics (such as duration, frequency, and intensity) were performed to explore the sources of heterogeneity in exercise interventions.

## 3. Results

### 3.1. Literature inclusion criteria

**Figure 1.**
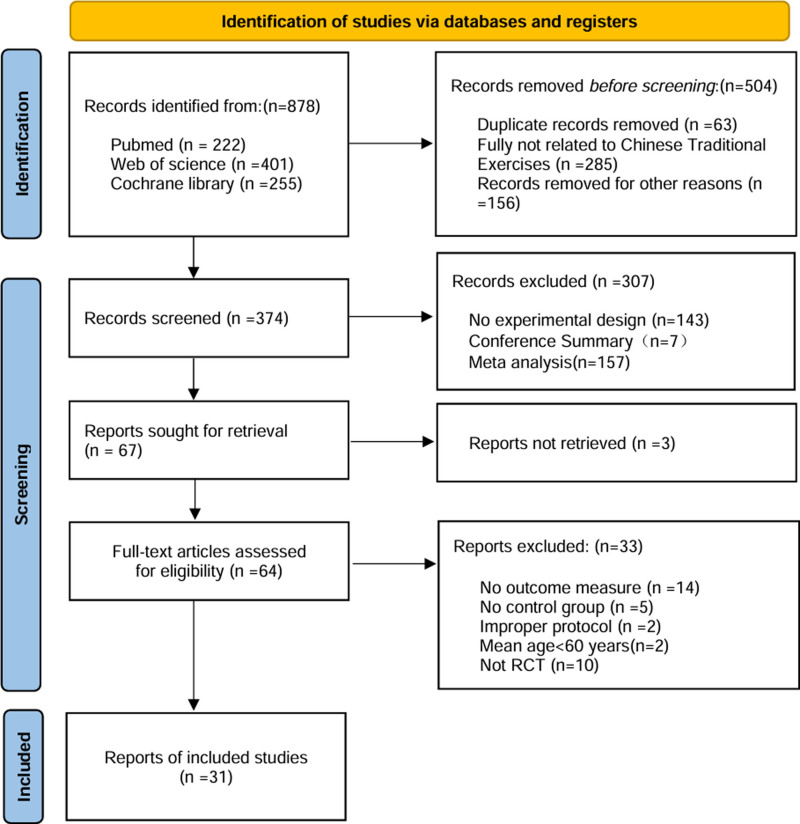
The process of selection of the eligible studies. RCT, randomized-controlled trial.

The literature selection process is illustrated in Figure 1. Initially, we retrieved 878 articles from electronic databases. In the first step, we excluded duplicate articles and studies not related to TCEs for various reasons, resulting in 374 articles remaining. In the 2nd step, we eliminated articles lacking experimental designs, conference abstracts, or those not relevant to the meta-analysis. Among the remaining 67 articles, 3 were unavailable in full text, leading us to 64 articles for comprehensive reading. During this process, we excluded 33 articles due to reasons such as lack of outcome measurements, absence of a control group, noncompliance with guidelines, or not being randomized-controlled trials. Ultimately, we identified 31 randomized-controlled trials that met the inclusion criteria and selected them for the meta-analysis.

### 3.2. Literature inclusion study features

The characteristics of the included studies are detailed in Table 1. Among the 31 studies, 9 focused on anxiety, while 29 addressed depression. In the randomized-controlled trials involving traditional Chinese healthcare techniques, 23 studies^[14,17–38]^ compared Tai Chi with control groups (such as maintaining existing lifestyles, engaging in normal daily activities, low-intensity activities, or conventional treatments), while 8 studies^[39–46]^ compared Qigong with nonexercise groups (such as maintaining existing lifestyles, normal daily activities, or reading newspapers). The intervention periods for traditional Chinese healthcare techniques ranged from 3 weeks to 1 year, with frequencies of 1–7 sessions per week, each lasting between 20 and 120 minutes.

**Table 1:** Detailed characteristics of each of the included literature studies

References	Country	Sample size		Mean age years		Intervention		Frequency and period	Outcome measures	Outcome
		E	C	E	C	E	C			
Babaei Bonab and Parvaneh^[17]^	Iran	35	35	60–70		Tai Chi	Routine daily activities	Three 60-min sessions/12wk	DAS	Anxiety
Liu et al^[14]^	China	30	30	60.90 ± 4.28	61.72 ± 3.54	Tai Chi	Usual lifestyle	Three 60-min sessions/24wk	GDS	Depression
Ge et al^[18]^	China	32	33	70.16 ± 5.40	72.91 ± 6.61	Tai Chi	Routine daily activities	Three 60-min sessions/8wk	GDS	Depression
Song et al^[19]^	China	20	20	64.15 ± 8.56	64.15 ± 8.56	Tai Chi	Health education	Three 60-min sessions/12 wk	SAS, SDS	Anxiety Depression
Li et al^[20]^	China	163	163	63.61 ± 6.62	65.44 ± 5.79	Tai Chi	Physical exercise	Seven 60-min sessions/6 mo	SAS, SDS	Anxiety Depression
Redwine et al^[21]^	USA	24	23	63 ± 9	67 ± 7	Tai Chi	Usual care	Two 60-min sessions/16 wk	BDI-IA	Depression
Lam et al^[22]^	China	171	218	77.2 ± 6.3	78.3 ± 6.6	Tai Chi	Physical exercise	Three 30-min sessions/1 yr	CSDD	Depression
Hsu et al^[23]^	China	30	30	80.7 ± 9.68	81.77 ± 6.32	Tai Chi	Routine daily activities	Three 40-min sessions/26 wk	GDS	Depression
Huang et al^[24]^	China	36	38	81.9 ± 6.0	81.9 ± 6.1	Tai Chi	Usual care	Three 20-min sessions/10 mo	GDS	Depression
Chou et al^[25]^	China	7	7	72.6 ± 4.2		Tai Chi	Usual care	Three 45-min sessions/3 mo	CES-D	Depression
Ma et al^[26]^	China	79	79	70.24 ± 0.25	69.7 ± 10.84	Tai Chi	Usual care	Three to five 90-min sessions/6 mo	CES-D	Depression
Leung et al^[27]^	Australia	19	19	73 ± 8		Tai Chi	Usual care	Five 30-min sessions/12 wk	HADS	Anxiety Depression
Yeh et al^[28]^	American	61	31	68.6 ± 9.2	68.1 ± 6.7	Tai Chi	Health education	Three 30-min sessions/12 wk	CES-D	Depression
Yildirim et al^[29]^	Turkey	30	30	62.9 ± 6.5	64.4 ± 7.5	Tai Chi	Physical exercise	Three 1-h sessions/12 wk	GDS	Depression
Solianik et al^[30]^	Lithuania	15	15	≥60		Tai Chi	Usual care	Two 60-min sessions/10 wk	HADS	Anxiety Depression
Irwin et al^31]^	USA	48	25	66.3 ± 7.4	66.4 ± 7.7	Tai Chi	Sleep seminar education	120 min/wk for 4 mo	IDS-C	Depression
Kilpatrick et al^[32]^	USA	21	19	67.1 ± 7.4	68.2 ± 5.4	Tai Chi	Health education	One 60-min sessions/12 mo	HAMD, GDS	Anxiety Depression
Lavretsky et al^[33]^	USA	89	89	69.2 ± 6.9	69.4 ± 6.2	Tai Chi	Health Education	60 min/wk for 12 wk	HAM-D	Depression
Chan et al^[34]^	China	24	24	75.4 ± 5.9	79.4 ± 8.5	Tai Chi	Usual care	Two 60-min for 3 mo	HAM-D	Depression
Lavretsky et al^[35]^	Turkey	36	37	69.1 ± 7.0	70.0 ± 7.4	Tai Chi	Health Education	One 2 h sessions/10 wk	HADS	Anxiety Depression
Yeh et al^[36]^	USA	8	8	68 ± 11	63 ± 11	Tai Chi	Aerobic exercise	Two 1-h sessions/12 wk	POMS scale	Depression
Frye et al^[37]^	The Netherlands	23	21	69.2 ± 9.26	69.2 ± 9.26	Tai Chi	Memory enhancement training	One 40-min session/12 wk	CES-D	Depression
Noradechanunt et al^[38]^	Australia	13	31	67.2 ± 8.3	65.2 ± 6.7	Tai Chi	Physical exercise	Two 90-min sessions/24 wk	CES-D	Depression
Tsang et al^[39]^	China	21	17	79.67 ± 6.55	80.65 ± 4.36	Qigong	Usual care	Three 45-min sessions/12 wk	HRSD	Depression
Lee et al^[40]^	China	14	14	≥60	≥60	Qigong	Cognitive training	Twice per wk/12 wk	DASS-A	Depression
Sun et al^[41]^	China	30	30	62.03 ± 7.37	65.23 ± 6.29	Qigong	Physical exercise	Seven 40-min sessions/3 wk	HAMD-24	Depression
Carcelén-Fraile et al^[42]^	Spain	57	60	69.70 ± 6.15	69.75 ± 6.76	Qigong	Routine daily activities.	Two 60-min sessions/12 wk	HADS	Anxiety Depression
Martínez et al^[43]^	Spain	29	29	76.1 ± 8.1	72.5 ± 8	Qigong	Usual care	Two 90-min session 7/4 wk	GDS	Depression
Jing et al^[44]^	China	39	40	75.25 ± 6.819	75.08 ± 5.264	Qigong	Cognitive training	Two 1–1.5 h/mo for 3 mo	GDS-15	Depression
Zhang et al^[45]^	China	21	24	67.48 ± 5.05	67.63 ± 5.17	Qigong	Usual care	One 30-min session/12 wk	SAS	Anxiety
Roswiyani et al^[46]^	Indonesia	67	65	71.9 ± 8.57	74.31 ± 9.57	Qigong	Routine daily activities	Two 90-min sessions/8 wk	BDI-II	Depression

BDI-IA = The 21-item Beck Depression Inventory−1A, C = control group, CES-D = Center for Epidemiologic Studies Depression Scale, CMI = Cornell Medical Index, CSDD = Cornell Scale for Depression in Dementia, DAS = death anxiety scale, DASS-A = depression anxiety stress scale-anxiety, E = experiment group, GDS = Geriatric Depression Scale, HADS = Hospital Anxiety and Depression Scale, HAMA = Hamilton Rating Scale of Anxiety, HAMD = Hamilton Rating Scale of Depression, HRSD = Hamilton Rating Scale of Depression, IDS-C = Inventory of Depressive Symptomatology, SAS = Self-rating Anxiety Scale, SDS = Self-rating Depression Scale.

Of the 31 included articles, 6 were from the United States, 15 were conducted in China, and 2 each were from Turkey, Australia, and Spain, while Lithuania, the Netherlands, Ireland, and Vietnam contributed 1 study each. The publication dates of these studies spanned from 2007 to 2022, with sample sizes ranging from a minimum of 14 to a maximum of 389. All participants were 60 years or older, with average ages ranging from 60.00 to 81.90 ± 6.10 years.

In terms of outcome measurement, the included studies employed a variety of assessment tools. Not only were there instruments specifically designed to measure anxiety and depression separately, but also the Hospital Anxiety and Depression Scale, which assesses both anxiety and depression simultaneously. Consequently, the tools used to evaluate anxiety and depressive symptoms varied across these studies.

### 3.3. Risk assessment

**Figure 2.**
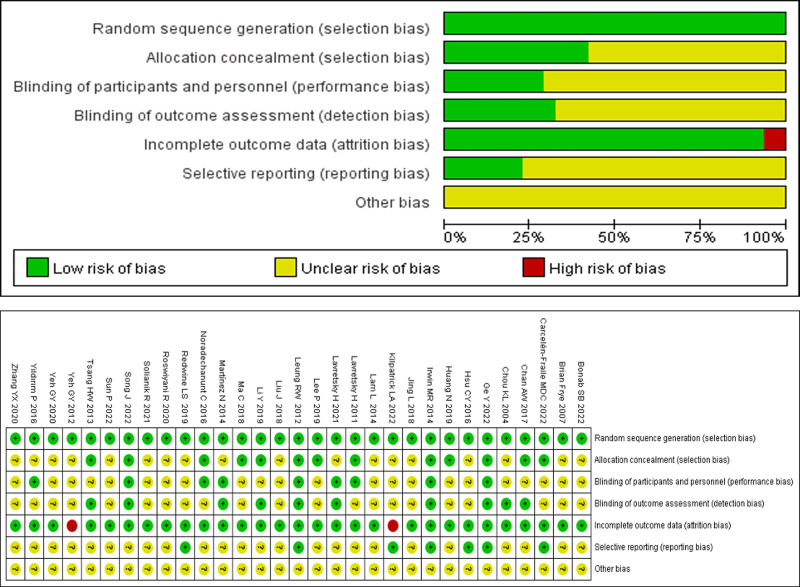
Quality assessment of included studies.

Figure 2 illustrates the risk of bias across the included studies. Among the 31 studies, random allocation was reported. Most studies detailed specific randomization methods, such as using random number tables, computers, or random sampling techniques. However, 7 studies did not specify the randomization method used. Only 13 studies reported employing envelope methods or centralized allocation (e.g., phone notifications) for concealed allocation, while the remaining 18 studies did not mention any form of concealed allocation. Most included studies did not employ blinding for participants and implementers, as it is challenging to blind individuals in TCE interventions. Only 9 studies explicitly described the blinding of outcome assessors. Regarding attrition bias, among the 31 trials, 2 studies that reported reasons for participant dropout were considered low risk, while 2 studies that did not report dropout reasons were deemed high risk. Seven studies provided clinical registration numbers, while 24 did not mention any registration numbers, leading to uncertainty regarding other bias assessments, which were generally classified as unclear risks. Overall, the reporting of bias across the 31 studies was moderate.

### 3.4. The effect of TCE on anxiety and depression of older adults

**Figure 3.**
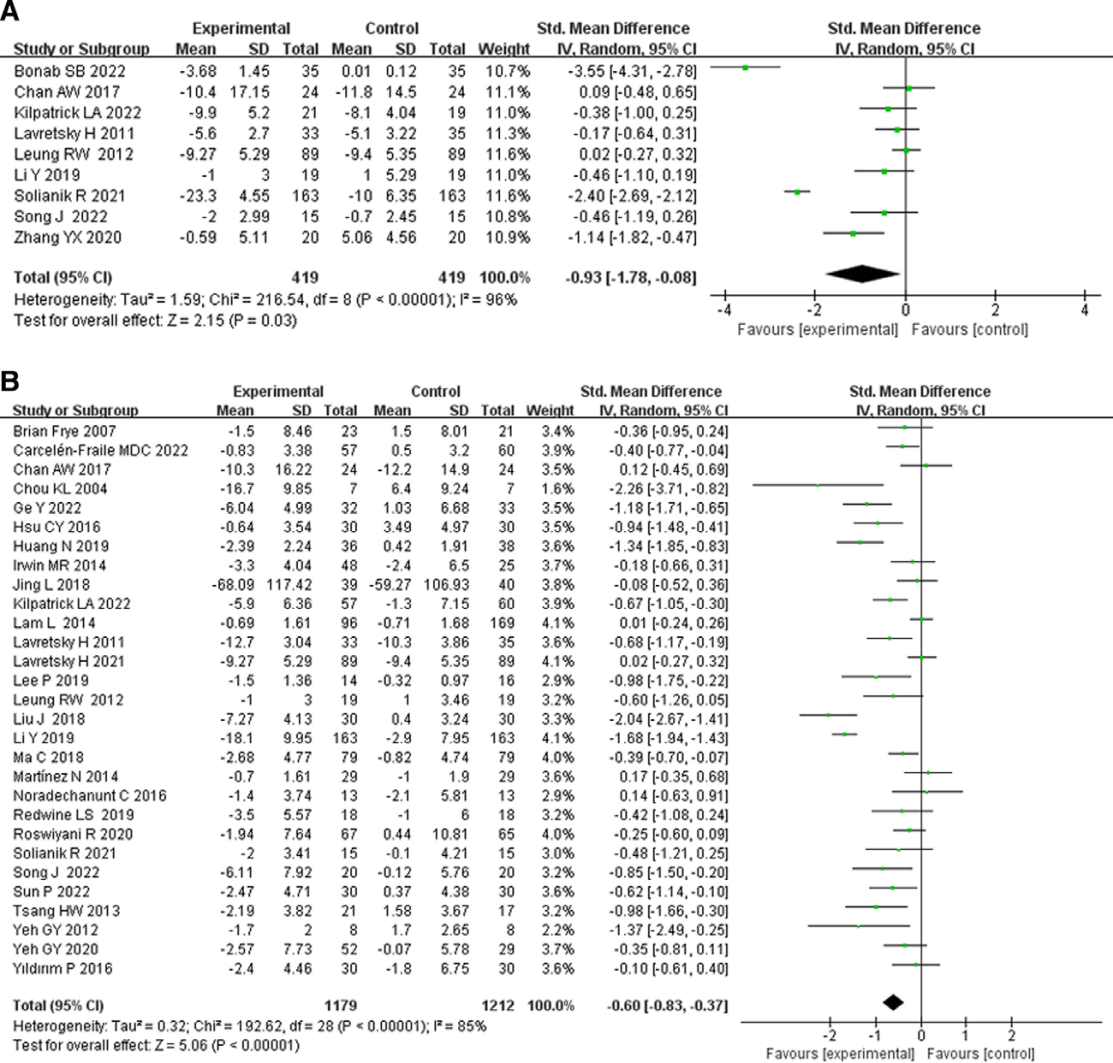
The effect of traditional Chinese exercise on (A) anxiety and (B) depression of old adults. CI = confidence interval, IV = inverse variance.

A total of 31 studies involved 2501 participants (1234 in the experimental group and 1267 in the control group) and assessed the impact of TCE on anxiety and depression in older adults. Results from 9 studies involving 774 participants indicated that TCE significantly improved anxiety scores (SMD = −1.14, 95% CI: −1.82 to −0.47, *P* = .03, *I*^2^ = 96%; Fig. 3A). Conversely, the combined results of 29 studies involving 2391 participants showed that TCE had a positive effect on improving depression scores (SMD = −0.60, 95% CI: −0.61 to −0.37, *P* < .00001, *I*^2^ = 85%; Fig. 3B).

### 3.5. Subgroup analysis results

#### 3.5.1. Effects of TCE on anxiety and depression in older adults by intervention duration

**Figure 4.**
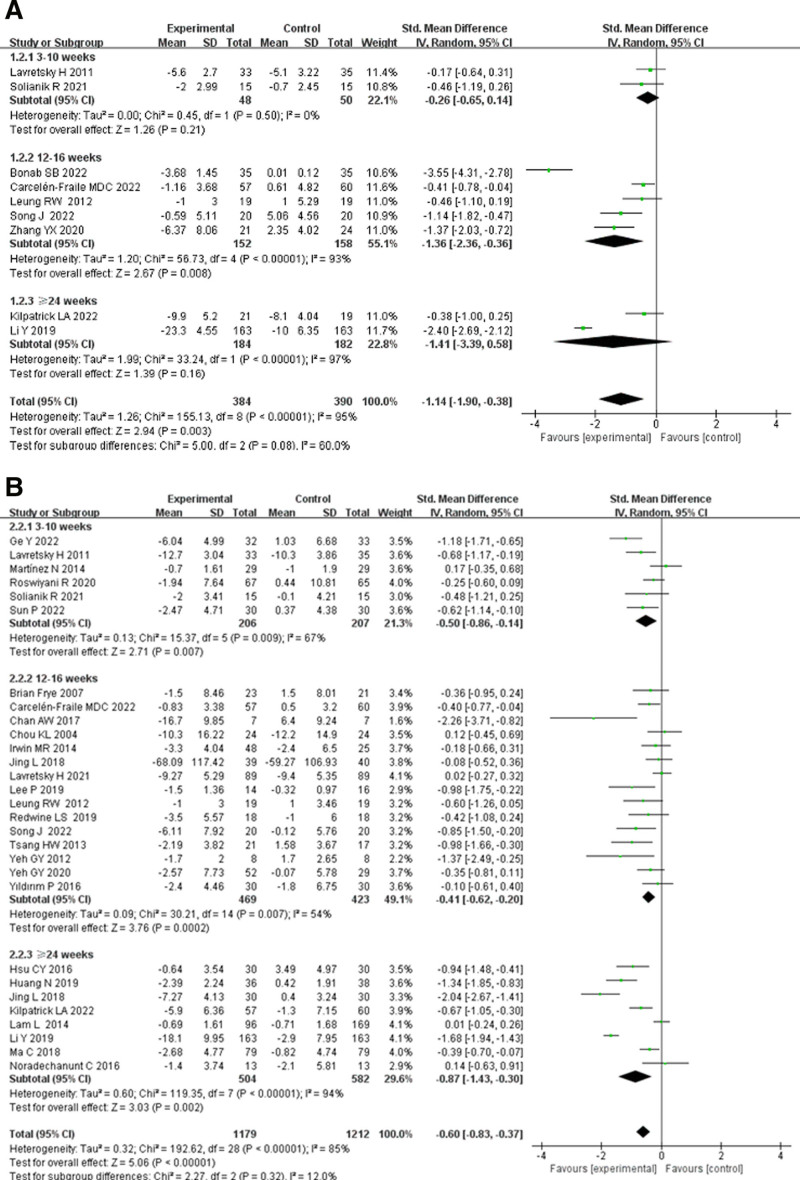
Effects of traditional Chinese exercise on (A) anxiety and (B) depression in old adults by intervention duration. CI = confidence interval, IV = inverse variance.

In the subgroup analysis of intervention duration, participants were divided into 3 groups: 3 to 10, 12 to 16, and over 24 weeks, to assess the impact of different intervention durations on anxiety and depression in older adults (Fig. 4).

As shown in Figure 4A, the impact of different intervention durations of TCEs on anxiety in older adults was evaluated. The anxiety subgroup included 774 participants, and there was high heterogeneity between the effect sizes of the 3 groups (*I*^2^ = 95%), indicating a significant relationship between intervention duration and the effect of TCEs on anxiety in older adults. In the 3–10 weeks group, the overall effect size was SMD = −0.26 (95% CI: −0.65 to 0.14, *P* = .21), which was not statistically significant. The 12 to 16 weeks group had the largest overall effect size (SMD = −1.36, 95% CI: −2.36 to −0.36, *P* = .008), showing a statistically significant difference. Finally, the 24-week group had an overall effect size of SMD = −1.14 (95% CI: −3.39 to −0.38, *P* = .16), which was also not statistically significant. These results suggest that an intervention duration of 12 to 16 weeks yields the best improvement in anxiety symptoms in older adults.

As shown in Figure 4B, the impact of different intervention durations of TCEs on depression in older adults was assessed. The depression subgroup included 2391 participants, and the effect sizes exhibited high heterogeneity (*I*^2^ = 85%), indicating that intervention duration significantly influences the relationship between TCEs and depression in older adults. In the 3 to10 weeks group, the overall effect size was SMD = −0.50 (95% CI: −0.14, *P* = .007), showing a statistically significant difference. The 12 to 16 weeks group had an overall effect size of SMD = −0.41 (95% CI: −0.62 to −0.20, *P* = .0002), which was statistically significant. Finally, the 24-week group had the largest overall effect size (SMD = −0.87, 95% CI: −1.43 to −0.30, *P* = .002), also showing a statistically significant difference. These findings suggest that interventions lasting 3 to 16 weeks improve depression symptoms in older adults, with the most important effects seen in interventions lasting over 24 weeks.

#### 3.5.2. Effects of TCE on anxiety and depression in older adults by intervention frequencies

**Figure 5.**
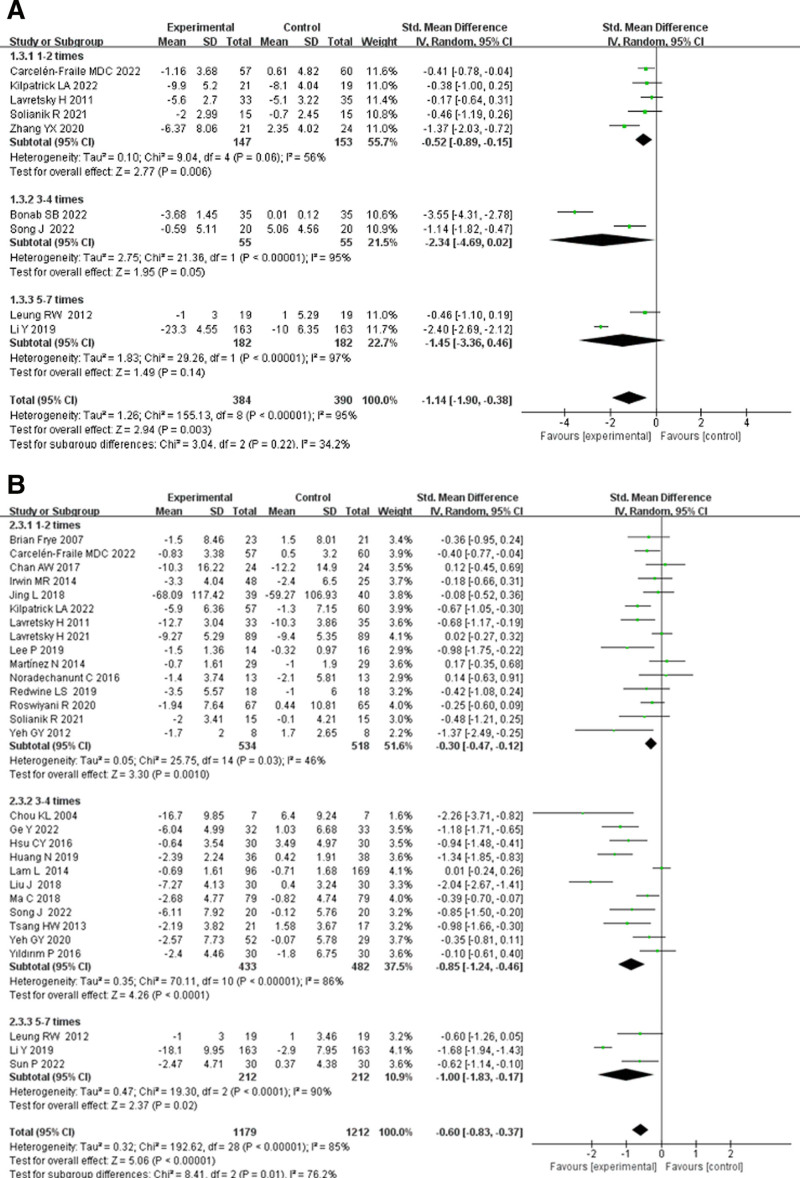
Effects of traditional Chinese exercise on (A) anxiety and (B) depression in old adults by intervention frequencies. CI = confidence interval, IV = inverse variance.

In the subgroup analysis of intervention frequency, participants were divided into 3 groups: 1 to 2, 3 to 4, and 5 to 7 times per week, to assess the impact of different frequencies of TCEs on anxiety and depression in older adults (Fig. 5).

As shown in Figure 5A, the impact of different intervention frequencies of TCEs on anxiety in older adults was assessed. The anxiety subgroup included 774 participants, and there was high heterogeneity between the effect sizes of the 3 groups (*I*^2^ = 95%), indicating a significant relationship between intervention frequency and the effect of TCEs on anxiety in older adults. In the 1 to 2 times per week group, the overall effect size was SMD = −0.52 (95% CI: −0.89 to −0.15, *P* = .006), showing statistical significance. The 3 to 4 times per week group had the largest overall effect size (SMD = −2.34, 95% CI: −4.69 to 0.02, *P* = .05), demonstrating a statistically significant difference. Finally, the 5 to 7 times per week group had an overall effect size of SMD = −1.45 (95% CI: −3.36 to 0.46, *P* = .14), which was not statistically significant. These results suggest that intervention frequencies of 1 to 4 times per week improve anxiety in older adults, while frequencies exceeding 5 to 7 times per week do not show significant effects.

As shown in Figure 5B, the impact of different intervention frequencies of TCEs on depression in older adults was examined. The depression subgroup included 2391 participants, and the effect sizes again exhibited high heterogeneity (*I*^2^ = 85%), indicating that intervention frequency significantly affects the relationship between TCEs and depression in older adults. In the 1 to 2 times per week group, the overall effect size was SMD = −0.30 (95% CI: −0.47 to −0.12, *P* = .001), showing a statistically significant difference. The 3 to 4 times per week group had an overall effect size of SMD = −0.85 (95% CI: −1.24 to −0.46, *P* < .0001), which was also statistically significant. Finally, the 5 to 7 times per week group had the largest overall effect size (SMD = −1.00, 95% CI: −1.83 to −0.17, *P* = .02), also showing statistical significance. This study suggests that intervention frequencies of 1 to 7 times per week all improve depression symptoms in older adults, with the best effects observed at frequencies of 5 to 7 times per week.

#### 3.5.3. Effects of TCE on anxiety and depression in older adults by single-session exercise

**Figure 6.**
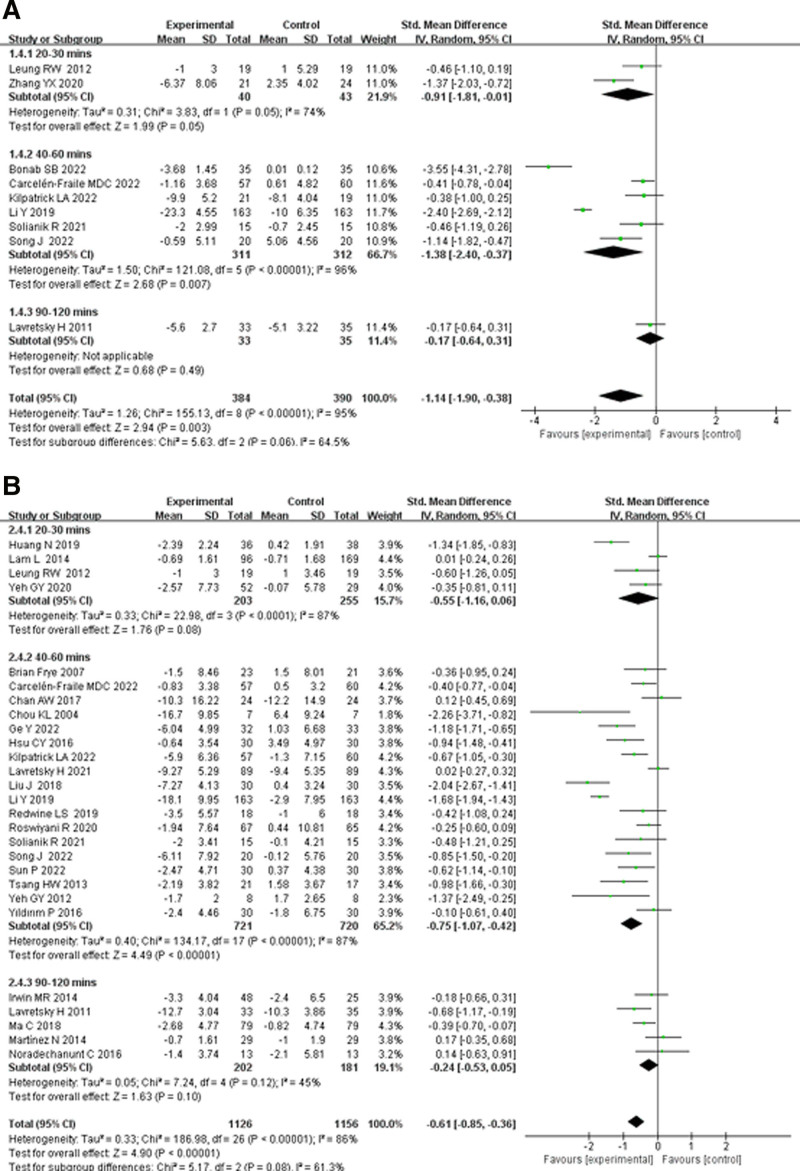
Effects of traditional Chinese exercise on (A) anxiety and (B) depression in old adults by single-session exercise. CI = confidence interval, IV = inverse variance.

In the subgroup analysis based on single-session exercise duration, participants were divided into 3 groups: 20 to 30, 40 to 60, and 90 to 120 minutes. This analysis assessed the effects of different exercise durations of TCEs on anxiety and depression in older adults (Fig. 6).

As shown in Figure 6A, the impact of different single-session exercise durations of TCEs on anxiety in older adults was examined. The anxiety subgroup included 774 participants, and there was high heterogeneity between the effect sizes of the 3 groups (*I*^2^ = 95%), indicating that single-session exercise duration significantly affects the relationship between TCEs and anxiety in older adults. The overall effect size in the 20 to 30 minutes group was SMD = −0.91 (95% CI: −1.81 to −0.01, *P* = .05), showing a statistically significant difference. The 40 to 60 minutes group had the largest overall effect size (SMD = −1.38, 95% CI: −2.40 to −0.37, *P* = .007), which was statistically significant. In contrast, the 90 to 120 minutes group had an overall effect size of SMD = −0.17 (95% CI: −0.64 to 0.31, *P* = .49), which was not statistically significant. These results suggest that single-session durations of 20 to 60 minutes improve anxiety in older adults, whereas the 90 to 120 minutes group showed no effect.

As shown in Figure 6B, the impact of different single-session exercise durations of TCEs on depression in older adults was assessed. The depression subgroup included 2391 participants, and there was high heterogeneity in the effect sizes (*I*^2^ = 86%), indicating that single-session exercise duration significantly affects the relationship between TCEs and depression in older adults. In the 20 to 30 minutes group, the overall effect size was SMD = −0.55 (95% CI: −1.16 to 0.06, *P* = .08), which did not reach statistical significance. The 40 to 60 minutes group had the largest overall effect size (SMD = −0.75, 95% CI: −1.07 to −0.42, *P* < .00001), which was statistically significant. Finally, the 90 to 120 minutes group had an overall effect size of SMD = −0.24 (95% CI: −0.53 to 0.07, *P* = .10), which was not statistically significant. These results suggest that single-session durations of 20 to 30 and 90 to 120 minutes do not improve depression in older adults, while only the 40 to 60 minutes group showed significant improvement.

Subgroup analysis results show that TCEs have significant effects in both anxiety and depression interventions, but the optimal intervention protocols differ. For anxiety interventions, the optimal protocol is 40 to 60 minutes per session, 3 to 4 times per week, for 12 to 16 weeks. For depression interventions, the best protocol is 40 to 60 minutes per session, 5 to 7 times per week, for 24 weeks or longer. These findings suggest that while Tai Chi and Qigong both have significant effects on alleviating anxiety and depression, anxiety interventions require a moderate exercise frequency, while depression interventions benefit from a higher frequency of practice to achieve the best results.

### 3.6. Exploration of sources of heterogeneity

To explore the sources of heterogeneity in the effects of anxiety and depression interventions, subgroup analyses were performed based on the control groups (Supplemental Figure S1, http://links.lww.com/MD/O551), intervention types (Supplemental Figure S2, http://links.lww.com/MD/O552), national populations (Supplemental Figure S3, http://links.lww.com/MD/O553), and measurement tools (Supplemental Figure S4, http://links.lww.com/MD/O554). In the anxiety analysis, heterogeneity was significantly reduced within the intervention types subgroups, showing a notable decrease compared to the overall analysis (Supplemental Figure S2, http://links.lww.com/MD/O552). However, for other factors, such as the control group, national population, and measurement tools, the changes in heterogeneity were minimal, suggesting that these factors have a relatively limited impact on the effects of anxiety interventions. In the depression analysis, heterogeneity was alleviated in the subgroups of control groups, intervention types, and measurement tools (Supplemental Figures S5, S6, and S8, http://links.lww.com/MD/O555, http://links.lww.com/MD/O557, and http://links.lww.com/MD/O562). This indicates that intervention type, control group, and measurement tools are key factors influencing the effects of depression interventions, while the different country variables have a relatively small impact (Supplemental Figure S7, http://links.lww.com/MD/O560).

### 3.7. Impact of different control groups on results

This study conducted a subgroup analysis comparing the effects of TCEs (Tai Chi and Qigong) on anxiety and depression in older adults. The studies were categorized based on the type of control group, which was divided into routine daily activity and low-intensity exercise groups. In the anxiety analysis, when the control group engaged in routine daily activities (such as health education, routine care, cognitive therapy, and sleep therapy), the effect size of TCEs was −0.64 (95% CI: −1.03 to −0.25), *P* = .04. In contrast, when the control group engaged in low-intensity exercise, the effect size was −2.90 (95% CI: −3.74 to −0.46), *P* < .00001 (Supplemental Figure S1, http://links.lww.com/MD/O551). For depression, when the control group engaged in routine daily activities, the effect size was −0.57 (95% CI: −0.81 to −0.33), *P* < .00001; and when the control group engaged in low-intensity exercise, the effect size was −0.63 (95% CI: −1.10 to −0.16), *P* < .00001 (Supplemental Figure S5, http://links.lww.com/MD/O555). These results indicate that TCE interventions are more effective in alleviating anxiety and depression when the control group engages in low-intensity exercise.

### 3.8. Publishing bias

**Figure 7.**
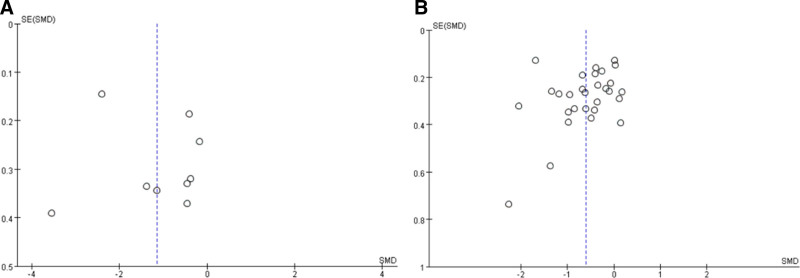
Publication bias of traditional Chinese exercise on (A) anxiety and (B) depression.

In conducting a systematic analysis of the 31 included studies, we comprehensively evaluated the effectiveness of various TCEs in alleviating anxiety and depression among older adults. The funnel plot generated using Review Manager 5.4 revealed a symmetrical distribution for both anxiety and depression (Fig. 7), indicating that there was no significant publication bias in the quality assessment. This finding further enhances the reliability and validity of the study results.

### 3.9. Sensitivity analysis

Excluding a specific study revealed that the influence of anxiety and depression on outcome indicators was restricted, failing to significantly alter the research findings. This underscores a degree of reliability and stability in the overall results.

## 4. Discussion

In recent years, increasing empirical evidence suggests that TCEs have a positive impact on alleviating anxiety and depression symptoms in older adults. However, there is currently a lack of systematic reviews that explore how the duration, frequency, and intensity of these exercises affect the mental health of old adults. Therefore, this study aimed to evaluate the intervention effects of TCEs such as Tai Chi and Qigong on anxiety and depression in older adults. This study included 31 studies with a total of 2501 participants, and the results showed that TCEs significantly improved anxiety and depression symptoms in older adults. Notably, subgroup analyses revealed that the duration, frequency, and intensity of exercise had varying effects on the intervention outcomes.

Medication and cognitive behavioral therapy (CBT) are proven treatments for anxiety and depression, but medication can lead to side effects and long-term dependence, and CBT requires face-to-face treatment,^[47]^ which is likely to result in patient resistance, whereas TCEs offer a noninvasive approach focusing on mental and physical well-being, resulting in reduced stress and enhanced emotional regulation. The TCE of Tai chi and Qigong focuses on slow-paced movements, deep breathing, and meditation to improve physical and mental health through physiological and psychological mechanisms. Despite similar core mechanisms, Tai Chi and Qigong differ in exercise intensity, goals, and regulatory mechanisms, which may affect intervention effectiveness.^[48,49]^ Tai Chi emphasizes balance and flexibility, improving muscle coordination and joint mobility through slow and flowing movements, with a strong focus on breath regulation and meditation. Tai Chi requires a high level of body control, particularly in terms of balance and coordination, and has a significant effect on enhancing flexibility, improving posture, and increasing muscle strength.^[50]^ For older adults, Tai Chi helps improve balance and reduce the risk of falls, which positively affects anxiety relief. In contrast, Qigong focuses more on breath regulation and the flow of “Qi,” emphasizing relaxation through deep abdominal breathing and meditation.^[51]^ The intervention focus of Qigong is to reduce mental stress and improve emotional states, which is especially effective for old adults prone to anxiety.

Despite their different intervention mechanisms, the results of this study show that both Tai Chi and Qigong significantly improve anxiety and depression symptoms in older adults. This suggests that both Tai Chi and Qigong share similar mechanisms of action by regulating the parasympathetic nervous system, promoting relaxation responses, and reducing the secretion of stress hormones to alleviate anxiety and depression. The combination of deep breathing and meditation helps to enhance emotional stability and self-regulation abilities.^[52,53]^ Our findings are consistent with previous studies, indicating that TCEs can significantly promote mental health in older adults.^[54–56]^ Specifically, Tai Chi shows great potential in alleviating anxiety. Physiologically, exercise helps relieve muscle tension, regulate neurotransmitter levels, and reduce the secretion of cortisol and other stress hormones,^[57,58]^ psychologically, exercise not only enhances physical activity levels and body regulation but also helps shift attention away from threats, reducing sensitivity to stressful stimuli and thereby improving self-esteem and self-efficacy.^[59–61]^

However, our findings differ from those of Song et al^[62]^ in their meta-analysis, which concluded that TCEs effectively alleviate depression but have limited effects on anxiety. This discrepancy may arise because their study included not only Tai Chi and Ba Duan Jin but also mindfulness interventions, which led to fewer studies on anxiety, resulting in different outcomes. Depression symptoms often impair the psychological and emotional health of older adults, especially with factors such as loneliness,^[63,64]^ illness, and bereavement exacerbating emotional distress, ultimately leading to depression.^[65,66]^ Therefore, providing appropriate recreational activities for old adults is crucial. As a safe, low-intensity activity, TCEs help mitigate age-related physical and cognitive decline, with Tai Chi being particularly effective in improving physical function, thereby positively influencing depression symptoms.^[67]^

This study found through subgroup analyses that there are significant differences in the intervention effects of TCEs on anxiety and depression, with optimal intervention protocols varying for each. In anxiety interventions, the best protocol is 40 to 60 minutes per session, 3 to 4 times per week, for 12 to 16 weeks. This protocol helps participants achieve physical and mental relaxation and balance through moderate session duration and frequency, especially as the combination of deep breathing and meditation activates the parasympathetic nervous system and reduces stress hormone levels, alleviating anxiety symptoms.^[68]^ In contrast, for depression, the study found that the most significant effect was achieved with 40 to 60 minutes per session, 5 to 7 times per week, for 24 weeks or longer. Compared with anxiety, depression symptoms may require higher exercise frequency to stabilize mood and enhance physical and psychological adaptability. Higher-frequency exercise can continuously promote brain plasticity and endocrine regulation and, through repetitive movement patterns, help alleviate negative emotions and improve psychological resilience.^[69]^ Both intervention protocols require at least 12 weeks, which is closely related to the physiological and psychological regulatory effects achieved through the long-term accumulation of TCEs. These findings guide practitioners: anxiety interventions can adopt moderate frequency (3–4 times per week), while depression interventions should use higher frequency (5–7 times per week) for optimal results.

In the anxious subanalysis, the study found that the intervention measures (Tai Chi and Qigong) were the main sources of heterogeneity. Subgroup analysis suggests that these 2 traditional forms of exercise have relatively consistent effects on anxiety intervention, which may be closely related to their common physiological mechanisms, such as regulating the autonomic nervous system through deep breathing and meditation, lowering stress hormone levels, and thereby alleviating anxiety symptoms.^[70]^ Furthermore, despite differences in control groups, national populations, and measurement tools, these factors did not significantly change heterogeneity, indicating that the intervention effects on anxiety are relatively stable across different research backgrounds. For depression indicators, subgroup analyses indicated that different interventions, different control groups, and different measurement tools played an important role in mitigating heterogeneity, and these findings provide ideas for the future in terms of designing and optimizing intervention programs.

Notably, we found that including cognitive training in the control groups significantly impacted the meta-analysis results. Cognitive training has been shown to enhance cognitive function, emotional regulation, and coping mechanisms, which are crucial for managing anxiety and depression.^[71]^ Therefore, cognitive training may significantly improve mental health, thus increasing the effect size in the control groups. Compared with cognitive training, low-intensity exercise primarily improves physical fitness.

Despite the valuable insights provided by this study, several limitations should be acknowledged. First, the majority of the interventions included in this meta-analysis were focused on Tai Chi, with comparatively fewer studies on Qigong. This imbalance may impact the generalizability and validity of the results. Second, due to the nature of exercise research, it was not feasible to blind participants or instructors, which could introduce potential bias due to differing expectations and behaviors between the experimental and control groups. Third, most of the studies relied on self-report questionnaires and lack of blinding, which may have affected the accuracy of the reported results. Finally, although databases like PubMed, Web of Science, and Embase cover most of the relevant literature, excluding databases such as Scopus may result in the omission of some studies.

Future research should include a broader range of high-quality studies and categorize participants based on age and health status to provide more tailored clinical guidance. In addition, using consistent intervention protocols and control group designs may help minimize potential biases in exercise interventions. Moreover, future studies should prioritize objective outcome measures, such as neurophysiological assessments, and implement double-blind designs to enhance data reliability. Furthermore, expanding the range of databases used would ensure a more comprehensive review of relevant studies. Finally, to build upon the findings of this systematic review, future research could benefit from exploring bibliometric analyses to identify emerging trends and research gaps in the field of TCEs and mental health interventions. Recent bibliometric studies have provided valuable insights into related areas such as sarcopenia research, sensor-based fall prevention, and geriatric rehabilitation, highlighting methodologies and trends that could inform the current research framework.^[72–75]^

### 4.1. Policy recommendations

This research highlights the significant positive impact of TCEs on alleviating anxiety and depression symptoms in older adults. By bolstering participants’ emotional well-being and diminishing negative feelings, these exercises play a crucial role in stress reduction and the management of anxiety-related conditions. Moreover, the convenience and cost-effectiveness of TCEs have made them increasingly popular. Hence, it is recommended that policymakers advocate for the introduction of tailored TCE programs for old adults, thus expanding their access to such activities. Furthermore, supportive measures should be put in place to promote and facilitate old adults’ engagement in TCEs, including the provision of education, training, and appropriate facilities. In addition, there is a need for government initiatives that integrate TCEs into existing healthcare strategies for older adults to optimize their mental health benefits.

### 4.2. Clinical practice

TCE has been acknowledged for its substantial advantages in mitigating anxiety and depression in old adults. In cases where older patients may not be viable candidates for or may decline pharmacological remedies, medical practitioners and healthcare experts are encouraged to suggest TCE as a viable mental health intervention. It is worth noting that the effectiveness of TCE can be impacted by various factors, including the length of the intervention, session frequency, and intensity levels. Hence, healthcare professionals are advised to design individualized TCE intervention strategies that are adapted to the unique needs of each patient to maximize positive mental health results.

## 5. Conclusions

The results of this study demonstrate that TCEs significantly improve anxiety and depression in older adults. Specifically, engaging in TCEs for 40 to 60 minutes per session, 3 to 4 times per week, over 12 to 16 weeks, effectively alleviates anxiety symptoms. In contrast, participating in physical activity 5 to 7 times per week, with sessions lasting 40 to 60 minutes each and sustained for more than 24 weeks, significantly improves depressive symptoms in older adults. Given the diversity in individual health conditions and preferences, clinicians should tailor exercise programs based on the patient’s age, health status, and personal preferences to maximize efficacy and adherence. Furthermore, policymakers should recognize the value of TCEs in improving the mental health of older adults and consider incorporating these exercises into community health programs.

## Author contributions

**Data curation:** Yangjian Dong, Dan Pang.

**Formal analysis:** Yangjian Dong, Jie Xiang, Guodong Chao.

**Methodology:** Yangjian Dong, Dan Pang.

**Writing – original draft:** Yangjian Dong, Dan Pang, Jie Xiang.

**Software:** Jie Xiang, Guodong Chao.

**Project administration:** Xiaoqin Kuang.

**Supervision:** Xiaoqin Kuang.

**Writing – review & editing:** Xiaoqin Kuang.

## Supplementary Material

SUPPLEMENTARY MATERIAL
