# Supplementary material for: Exploring the benefits of traditional Chinese exercises (Tai Chi and Qigong) on the anxiety and depression of older adults: A systematic review and meta-analysis
Source: Medicine (Baltimore). 2025 Mar 21;104(12):e41908. doi: 10.1097/MD.0000000000041908 (PMC11936652; doi:10.1097/MD.0000000000041908)
Supplement: SUPPLEMENTARY MATERIAL [file medi-104-e41908-s001.docx]

**Search strategy**

**web of science：**

| **#1** | (TS=(Aged)) OR TS=(elderly) |
| --- | --- |
| **#2** | ((((((((((((((((((TS=(Tai Ji)) OR TS=(Tai-ji)) OR TS=(Tai Chi)) OR TS=(Chi, Tai))) OR TS=(Tai Ji Quan)) OR TS=(Ji Quan, Tai)) OR TS=(Quan, Tai Ji)) OR TS=(Taiji)) OR TS=(Taijiquan)) OR TS=(T'ai Chi)) OR TS=(Tai Chi Chuan)) OR TS=(qigong))) OR TS=(Liu Zi Jue)) OR TS=(Wu Qin Xi)) OR TS=(Six Healing Sounds)) OR TS=(Ba Duan Jin)) OR TS=(Traditional Chinese exercise) OR TS=(Yi Jin Jing) |
| **#3** | ((TS=(randomized controlled trial)) OR TS=(controlled clinical trial)) OR TS=(randomized) |
| **#4** | ((((((((((((((TS=(Anxiety)) OR TS=(Angst)) OR TS=(“Social Anxiety”)) OR TS=(“Anxieties, Social”)) OR TS=(“Anxiety, Social”)) OR TS=("Social Anxieties")) OR TS=(Hypervigilance)) OR TS=(Nervousness)) OR TS=(Anxiousness)) OR TS=(Depression)) OR TS=("Depressive Symptoms")) OR TS=("Depressive Symptom")) OR TS=("Symptom, Depressive")) OR TS=("Emotional Depression")) OR TS=("Depression, Emotional") |
| **#5** | #1 AND #2 AND #3 AND #4 |
