# Supplementary figures and images for: Exploring the benefits of traditional Chinese exercises (Tai Chi and Qigong) on the anxiety and depression of older adults: A systematic review and meta-analysis
Source: Medicine (Baltimore). 2025 Mar 21;104(12):e41908. doi: 10.1097/MD.0000000000041908 (PMC11936652; doi:10.1097/MD.0000000000041908)

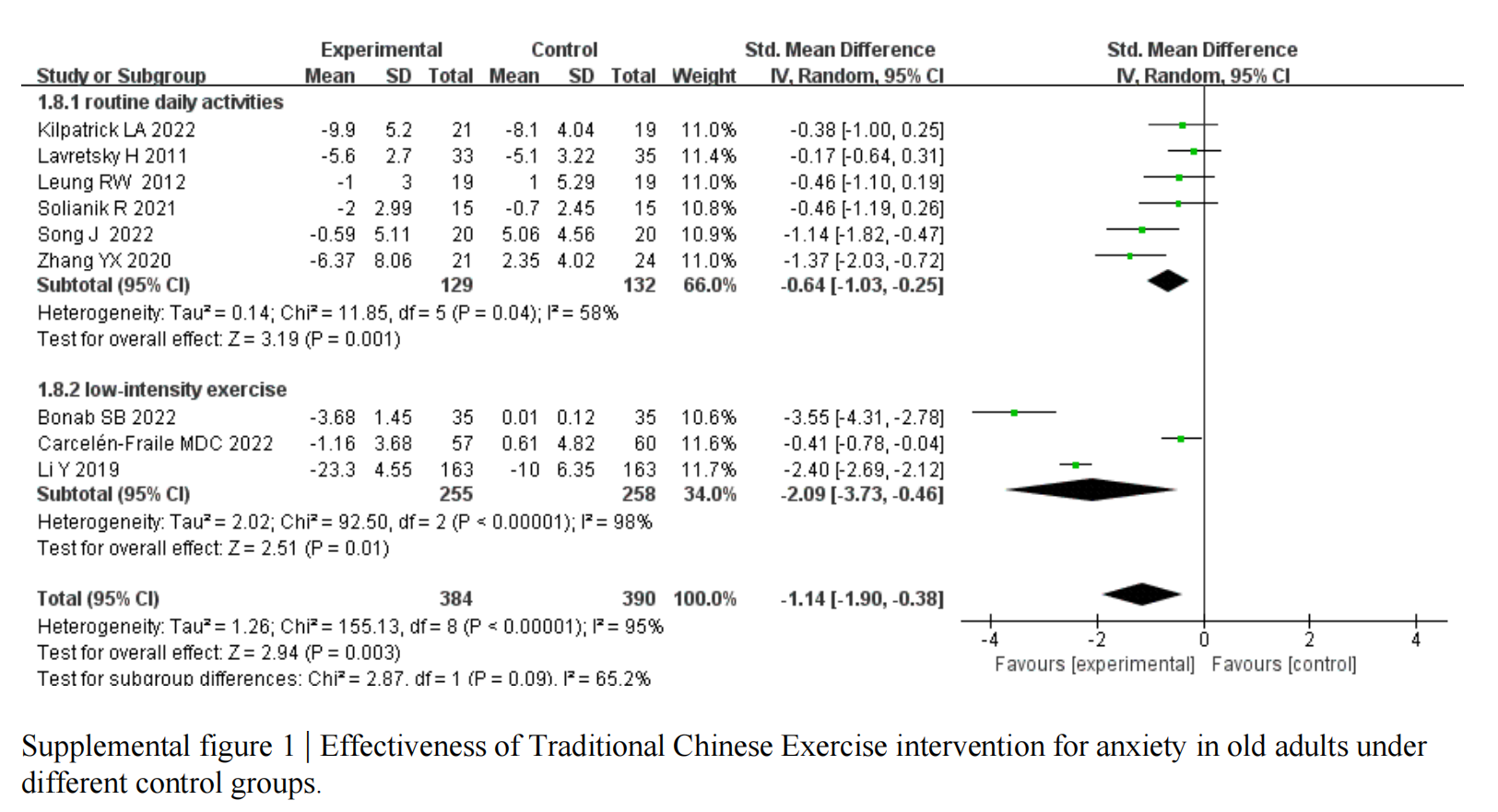

Supplement: SUPPLEMENTARY MATERIAL [file medi-104-e41908-s002.tif]

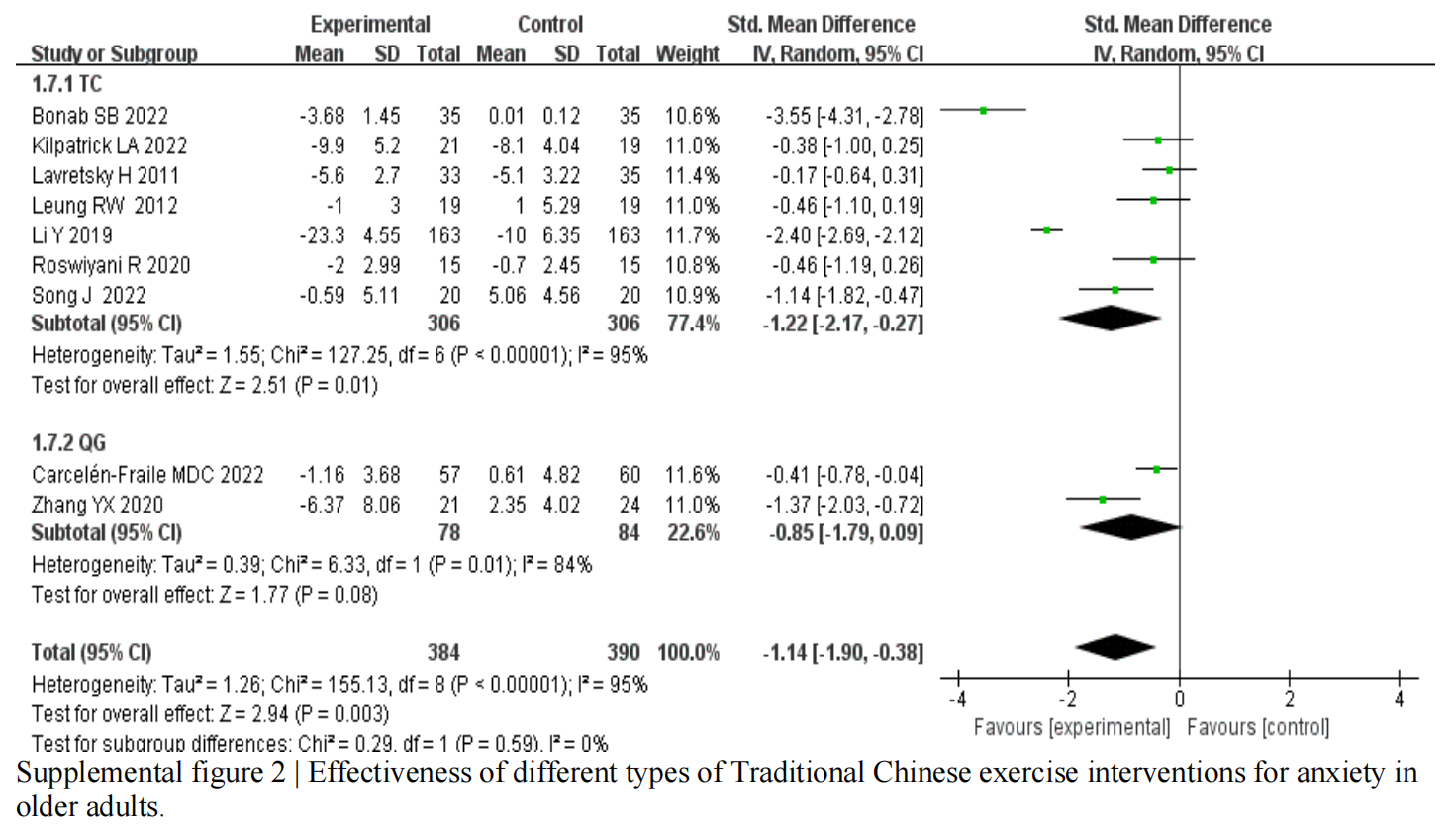

Supplement: SUPPLEMENTARY MATERIAL [file medi-104-e41908-s003.tif]

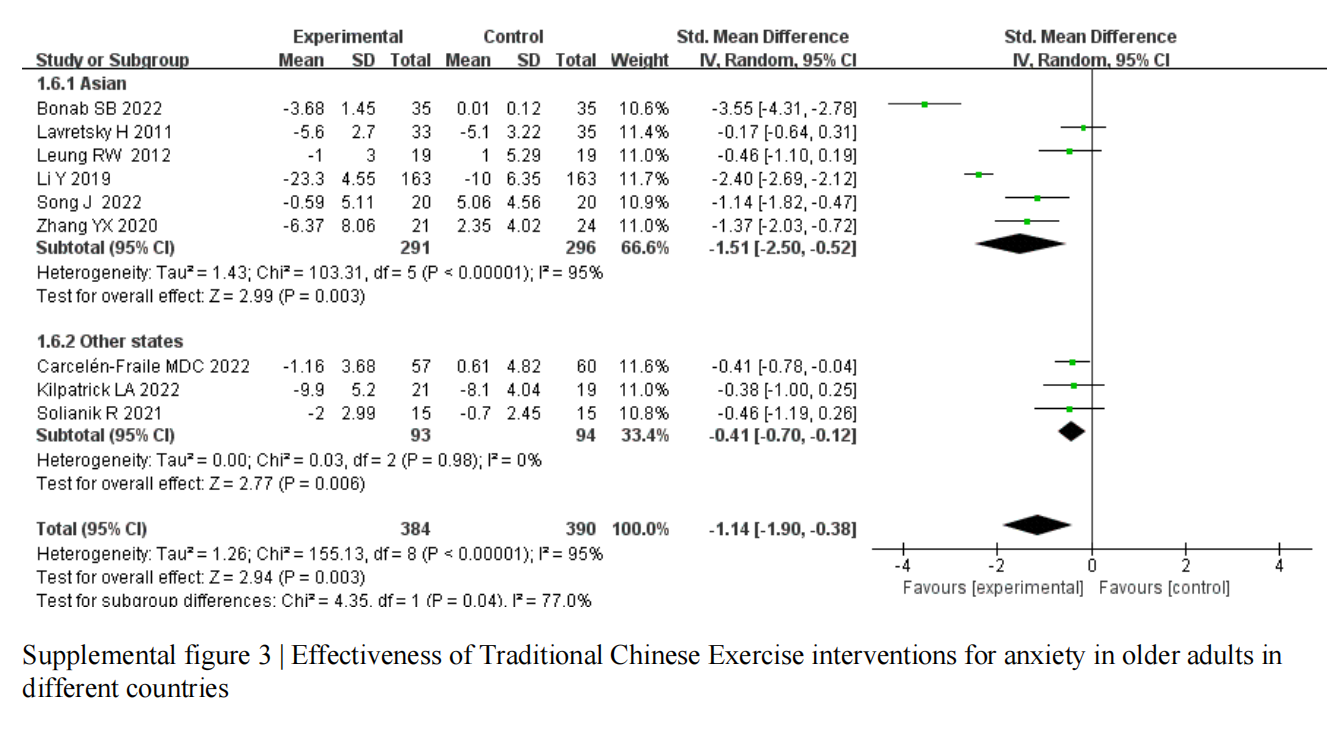

Supplement: SUPPLEMENTARY MATERIAL [file medi-104-e41908-s004.tif]

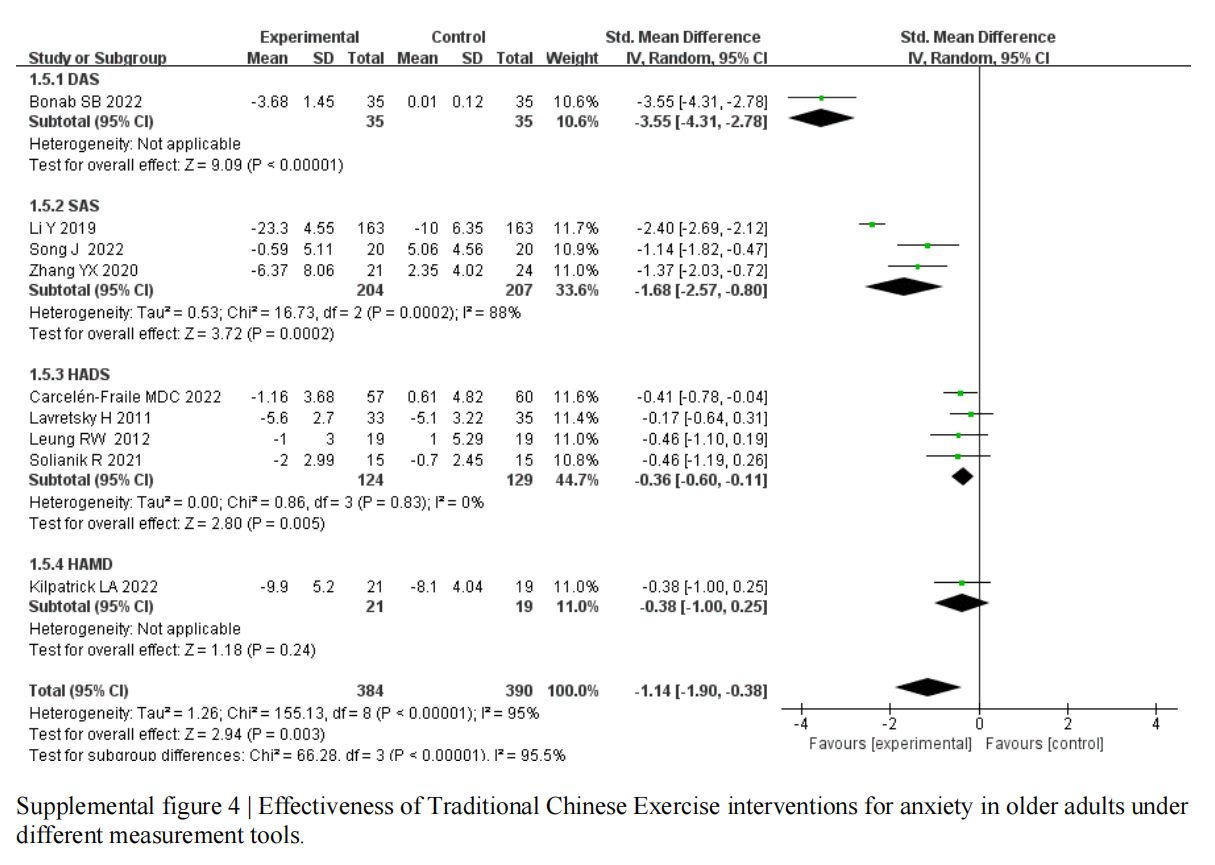

Supplement: SUPPLEMENTARY MATERIAL [file medi-104-e41908-s005.tif]

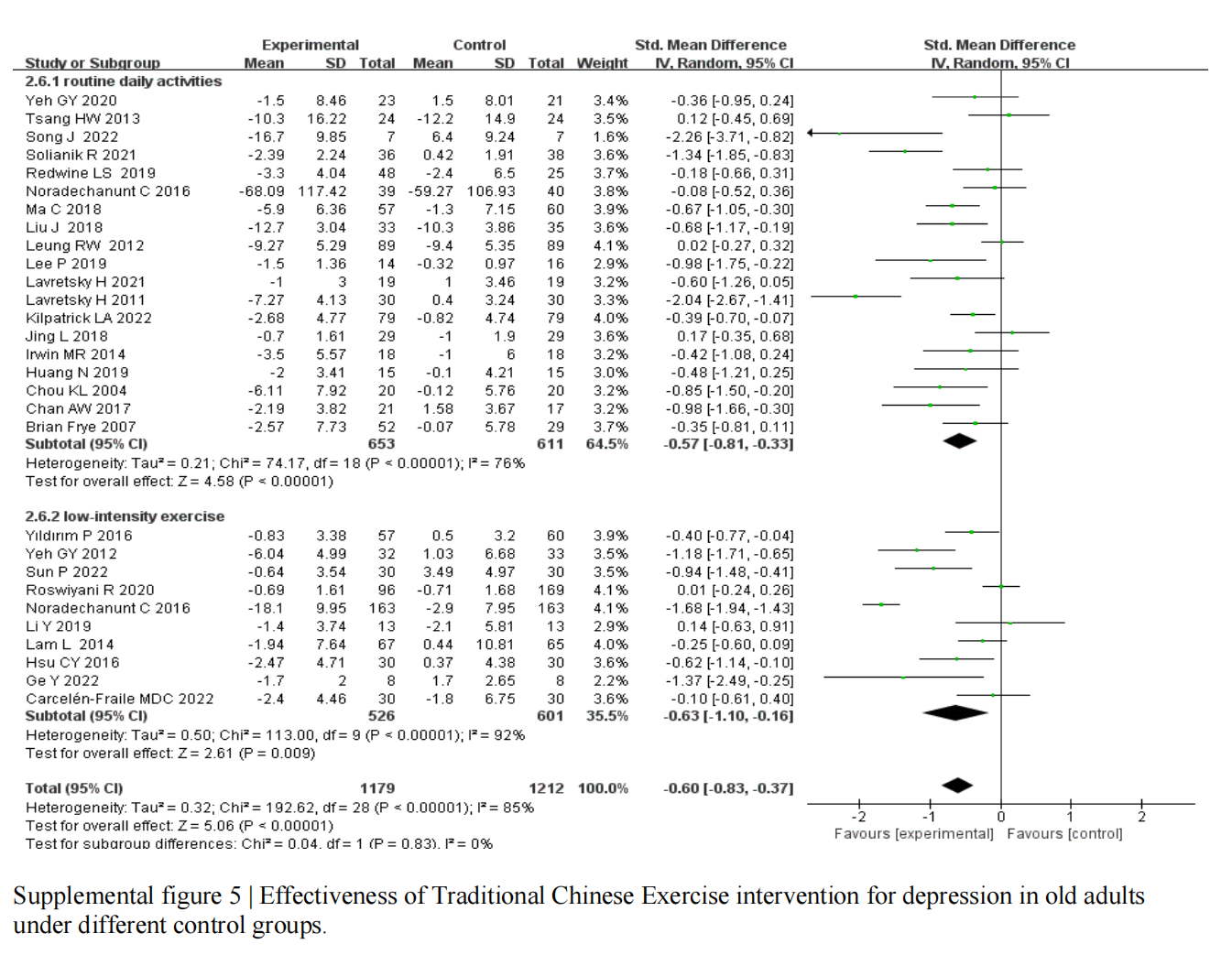

Supplement: SUPPLEMENTARY MATERIAL [file medi-104-e41908-s006.tif]

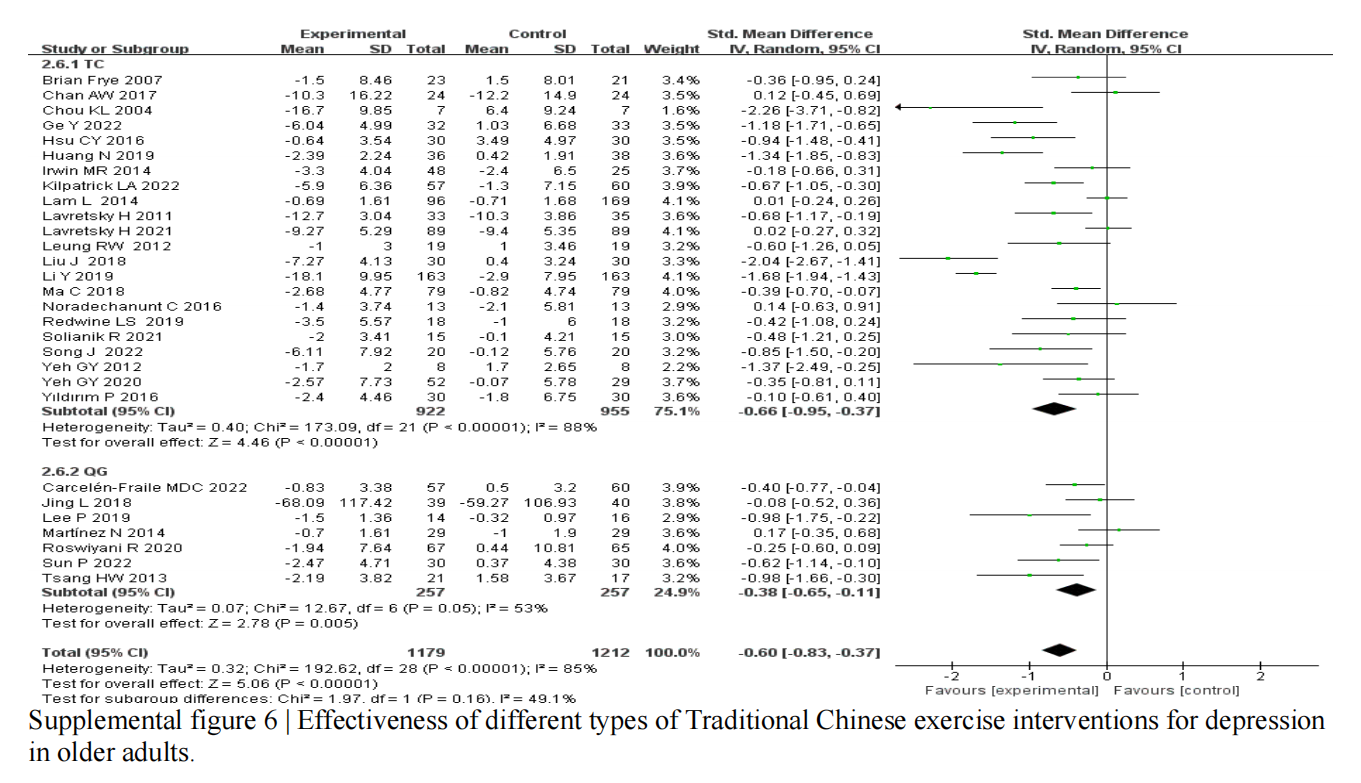

Supplement: SUPPLEMENTARY MATERIAL [file medi-104-e41908-s007.tif]

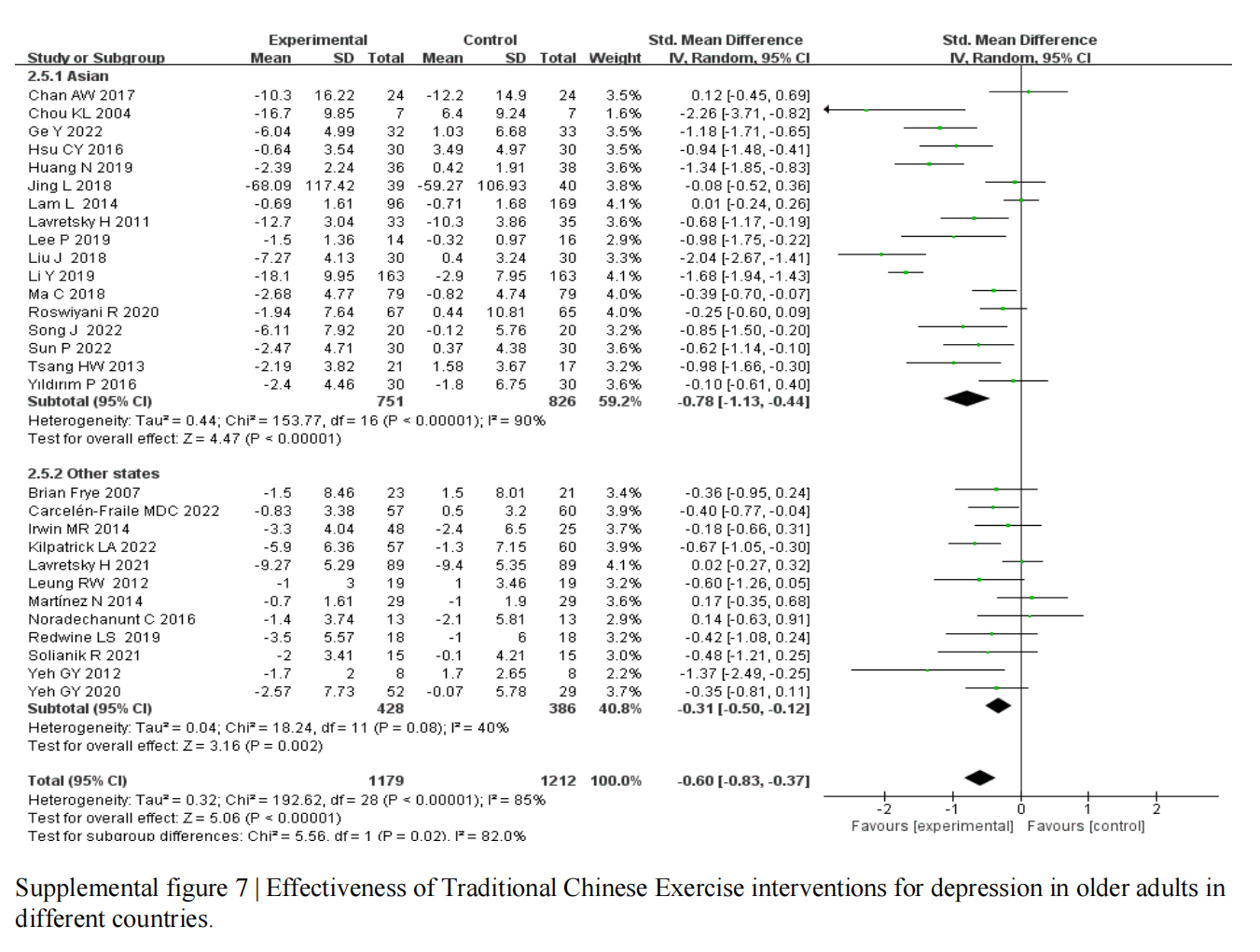

Supplement: SUPPLEMENTARY MATERIAL [file medi-104-e41908-s008.tif]
